# Insights from nanotechnology in COVID-19: prevention, detection, therapy and immunomodulation

**DOI:** 10.2217/nnm-2021-0004

**Published:** 2021-05-17

**Authors:** Priya Singh, Deepika Singh, Pratikshya Sa, Priyanka Mohapatra, Auromira Khuntia, Sanjeeb K Sahoo

**Affiliations:** ^1^Institute of Life Sciences, Bhubaneswar, Odisha, 751023, India; ^2^Regional Center for Biotechnology, Pali, Haryana, 121001, India

**Keywords:** COVID-19, diagnosis, immunomodulation, nanotechnology, therapy

## Abstract

The outbreak of SARS-CoV-2 infection has presented the world with an urgent demand for advanced diagnostics and therapeutics to prevent, treat and control the spread of infection. Nanotechnology seems to be highly relevant in this emergency due to the unique physicochemical properties of nanomaterials which offer versatile chemical functionalization to create advanced biomedical tools. Here, nano-intervention is discussed for designing effective strategies in developing advanced personal protective equipment kits, disinfectants, rapid and cost-effective diagnostics and therapeutics against the infection. We have also highlighted the nanoparticle-based vaccination approaches and how nanoparticles can regulate the host immune system against infection. Overall, this review discusses various nanoformulations that have shown clinical relevance or can be explored in the fight against COVID-19.

## COVID-19: A pandemic

**Figure 1.**
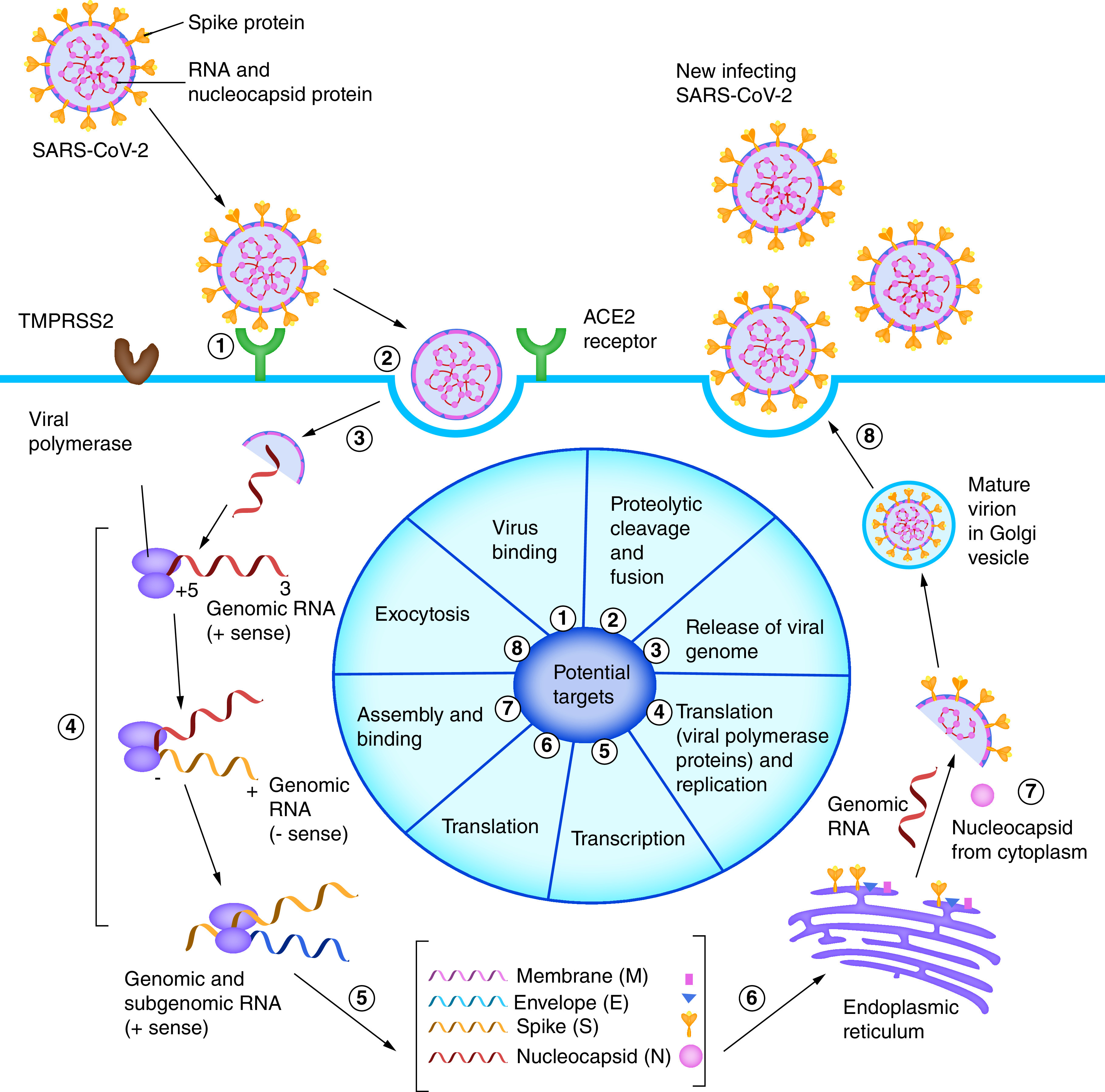
Life cycle and potential targeting sites of SARS-CoV-2. **1.** Attachment of SARS-CoV-2 viral S protein with ACE2 receptor present on the host cell membrane. **2.** Binding of viral S protein with the ACE2 receptor permits the entry of the virus into the host cell. **3.** After entry of the virus into the cell, the viral envelope undergoes proteolytic cleavage and releases its genomic RNA into the cytoplasm. **4,5.** The genomic RNA is then converted into smaller subgenomic mRNA, which is translated to S, E, M and other proteins that are required for the assembly of the virus. **6 and 7.** Next, the S, E and M proteins enter the endoplasmic reticulum (ER), followed by the formation of mature virion by their combination with nucleocapsid (N) protein (which is synthesized in the cytoplasm) and positive-strand genomic RNA in the ER–Golgi compartment. **8.** Finally, the completely formed virus particles are released out of the cells through exocytosis, to repeat the same cycle by infecting other cells.

The recent pandemic that has affected the globe is caused by a virus that was first detected in Wuhan (Hubei province, China) and named severe acute respiratory syndrome coronavirus 2 (SARS-CoV-2) on 11 February 2020 [1,2]. The WHO named the disease caused by SARS-CoV-2, coronavirus disease 2019 (COVID-19). Initial cases of the disease were reported as pneumonia of unknown origin, but after a detailed investigation, it was revealed that a type of coronavirus caused the disease [3]. The widespread human infection and transmission of SARS-CoV-2 triggered a high alert and forced the WHO to declare a pandemic in March 2020 [4]. SARS-CoV-2 is an enveloped virus with a positive-sense single-stranded RNA genome of 34 kb [5]. The first two-thirds of the genome encodes replicase genes, which are translated and processed into 15–16 nonstructural proteins. The other third consists of open reading frames for the four structural proteins – envelope (E), membrane (M), nucleocapsid (N) and spike (S) – which are essential for assembly and budding, maintaining integrity, forming nucleocapsid and enabling the attachment of the virus to host cells by binding to ACE2, respectively [5–7]. The expression of ACE2 determines the uptake of the virus in different tissues; as the respiratory tract has the highest expression of ACE2, it is the main target of SARS-CoV-2 [8]. The entire life cycle of SARS-CoV2 and the pathophysiology, along with potential targets for inhibition of the virus, are illustrated in Figure 1. COVID-19 is a very contagious disease and evidence suggests that SARS-CoV-2 can spread through direct, indirect or close contact with an infected person, mainly by inhaling virion particles contained in respiratory fluid droplets which are expelled through sneezing, coughing or talking [9]. Furthermore, as these viruses can be active for hours on inanimate surfaces like metals, papers, plastics and cloths depending on temperature, humidity and the chemical and topological nature of the solid surface, the infection can also be transmitted by touching these virus-laden surfaces and then touching eyes, nose or mouth [6]. Common symptoms of the disease include fever, dry cough and tiredness, while body pain, nasal congestion, headache, conjunctivitis, sore throat or diarrhea are also observed in some patients. Serious symptoms, like difficulty in breathing or shortness of breath, chest pain and loss of speech or movement are also observed in a few cases [10]. In addition to these, the evidence is increasing for neurological manifestations associated with SARS-CoV-2 infection; symptoms and syndromes like dizziness, ataxia, neuropathic pain, headache, myopathy, epilepsy and ischemic stroke are getting commonly manifested in severe COVID-19 patients. However, not all SARS-CoV-2-infected people show symptoms, and some asymptomatic patients act as carriers. However, it has been reported that these carriers have hyposmia (decreased sense of smell), which can act as a marker for identifying asymptomatic patients [11].

It is observed that people with a history of hypertension, diabetes, obesity, chronic lung disease or cardiovascular issues are at higher risk of SARS-CoV-2 infection and mortality. Further, patients aged >80 years are at a higher risk, with 14.8% mortality [12]. These give the impression that elderly people and those living with chronic diseases have higher mortality than healthy individuals when infected with SARS-CoV-2. At the time of writing (January 2021), SARS-CoV-2 has infected 105,470,677 people, among whom 2,296,064 have died due to the disease [13]. Further, so-called ‘second waves’ of the disease are happening, with mutant strains of SARS-CoV-2 spreading in some countries. Reports suggest that the mutant strain emerging in the UK is more infectious than the previous strain: each infected individual could infect an average of 1.5 other people, rather than the 1.1 average for the earlier strain. However, as of now, there is no indication as to whether it could cause more severe disease-related complications or deaths [14,15]. This continued rise of both cases and deaths, with the emergence of variant strains, necessitates the development of new treatment methodologies to control and treat the infection. Though we are in the age of the highest technological advancements and are well aware of the complete structural details of SARS-CoV-2, we are still struggling to develop a cure for the disease [16]. This is mainly because the development of new drugs requires a long approval process to prove their efficacy and safety, whereas the effectiveness of conventional antiviral treatments fades with the emergence of viral mutations. Thus multidisciplinary research efforts are quintessential to combat this pandemic [17]. In this context nanotechnology, which is an amalgamation of physics, chemistry, biology and engineering, can offer several advantages due to its unique physicochemical properties [18]. This review gives comprehensive detail on the advantages of employing nanotechnology in the design of diagnostic tools, vaccines, therapy and immunomodulation.

## Immunopathology & COVID-19

 The SARS-CoV-2 incubation period in the host varies from person to person; according to the WHO, it takes 2–14 days after the initial infection. People infected with SARS-CoV-2 often experience a local infection in cells that line the airways of the lungs, which in turn triggers the immune response to remove the virus and aid in recovery [19]. Pathological studies of SARS-CoV-2-infected patients showed the presence of both T cell and B cell immune responses. After the onset of infection, CD8^+^ T cells can directly kill the virus-infected cells, whereas CD4^+^ T cells prime both CD8^+^ T cells and B cells to generate an immune response. CD4^+^ T cells also drive the cytokine production that recruits other immune cells to the site of infection [20]. Activated B cells are also reported in the blood of SARS-CoV-2-infected patients. These activated B cells produce antibodies initially against the nucleocapsid (N) protein and then against the S protein of the virus to generate an immune response against it [21].

Cytokines play a key role in initiating and orchestrating the host immune response upon viral infection as an antiviral defense mechanism. Some cytokines can directly induce an antiviral state or apoptosis in virus-infected cells, while others can mediate the activation of the immune system to kill the infected cell. Another class of cytokine, known as chemokines, controls the traffic of immune cells migrating toward the site of infection or inflammation [22]. Several studies have reported the dysregulated immune response with relevant changes of both innate and adaptive immunity in SARS-CoV-2-infected patients. Modulation of total neutrophils along with a marked decline in the level of circulating CD4^+^ cells, CD8^+^ cells, B cells and natural killer cells is correlated with disease severity and death in COVID-19 patients [23–26]. Besides changes in blood cell counts, most patients with severe SARS-CoV-2 infection displayed a ‘cytokine storm’, as elevated levels of proinflammatory cytokines like IL-6, IL-1β, IL-2, IL-8, IL-17, G-CSF, GM-CSF, IP-10, MCP-1, CCL3 and TNF-α were observed in their serum [27]. Although a well-co-ordinated and rapid immune response is generated in patients which represents the first line of defense against SARS-CoV-2 infection, the excessive inflammatory innate response and dysregulated adaptive response may lead to severe tissue damage of the host, not only at the site of virus entry but also at the systemic level. This uncontrolled immune response is expected to result in acute lung injury and acute respiratory distress syndrome, which is a major concern for COVID-19 patients [24,28].

## Role of nanotechnology in the COVID-19 pandemic

**Figure 2.**
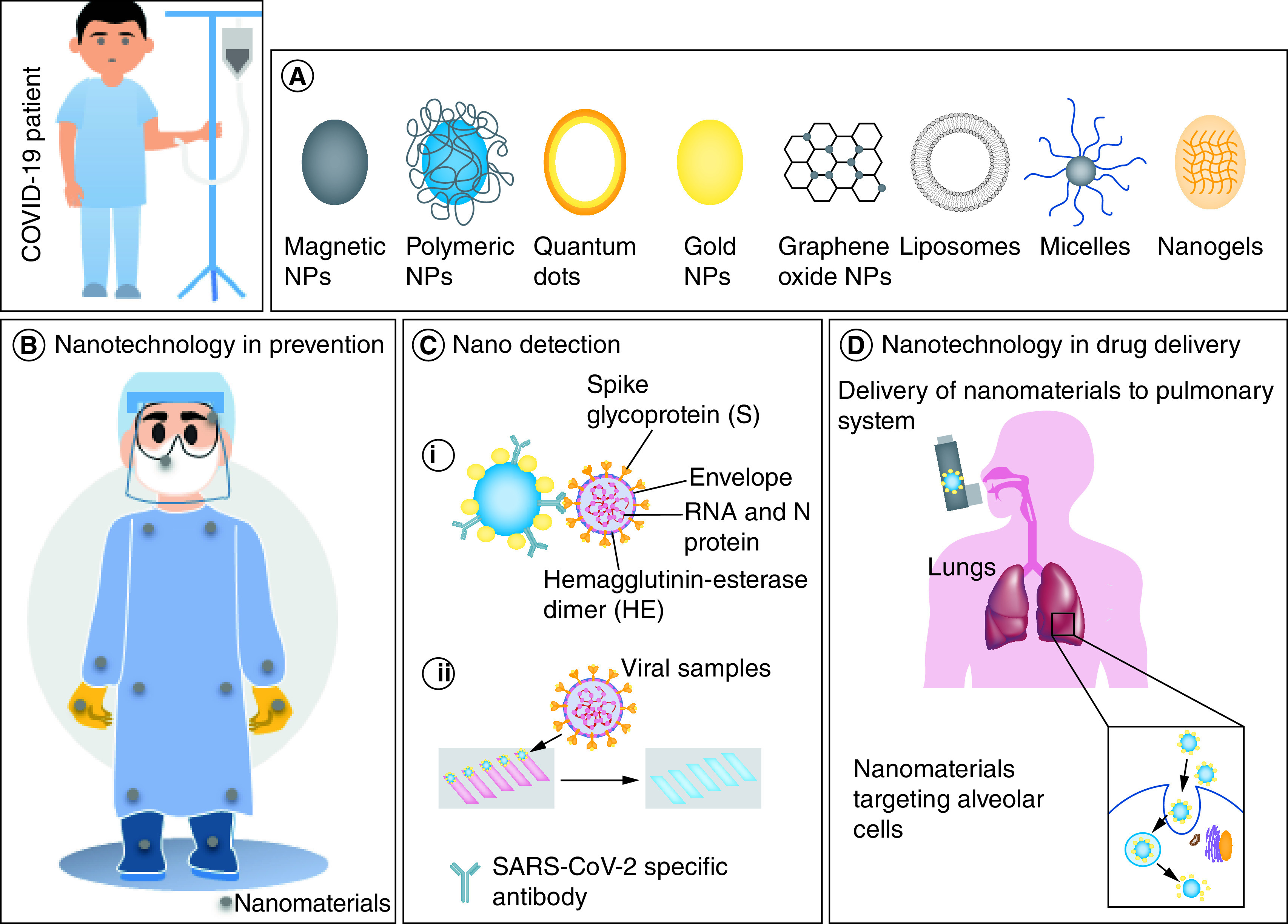
Nanotechnology-based approaches to combat COVID-19. **(A)** Different nanoparticles can be integrated for prevention, detection or therapy against SARS-CoV-2 infection. **(B)** NPs that can deactivate the virus can be used in the manufacture of face masks, face shields, safety glasses, shoe covers, disposable gloves and gowns, which are routinely being used by healthcare workers. **(C)** For detection, **(i)** NPs can be conjugated with SARS-CoV-2 specific antibody, which can emit fluorescence when it encounters the virus or **(ii)** nanoparticle-coated chips can be made, which have the capacity to change their color when infected samples are loaded on them. **(D)** NPs can also be used to deliver drugs directly into the virus-infected alveolar cells present in the lungs through inhalation. NP: Nanoparticle.

Nanotechnology is the design and application of materials one of whose dimensions is <100 nm [29]. It has made an immense contribution in many fields of science, including materials science, physics, chemistry, biology, engineering and computer science [30]. Recent years have witnessed the spur of nanotechnology in biomedical sciences, where it has been successfully employed for detection, diagnosis and treatment of diseases [31]. The widespread use of nanotechnology in medical sciences can be attributed to its unique properties like small size, large surface area, multifunctionality, surface adaptability and enhanced solubility which helps in the development of safer and more efficient drug candidates, tissue-targeted therapies, personalized medicines and early diagnostic devices. In the current COVID-19 pandemic situation, the potential of nanotechnology is unquestionable. It can be used in various spheres in the fight against COVID-19, such as prevention, diagnosis and therapy [32]. For preventing the spread of the virus, it can be used in the development of effective disinfectants and surface coatings, self-sterilizing personal protective equipment (PPE) for healthcare personnel, and infection-safe masks [17]. As COVID-19 is a highly infectious disease, it is essential to develop specific and sensitive sensors that can quickly identify the infection or immunological response for rapid point-of-care (POC) diagnostics, surveillance and monitoring of disease. Here nanotechnology, with its potential to develop simple, fast and cost-effective assays using gold-based nanoparticles and other inorganic nanoparticles, can be used to design assays to monitor the presence of SARS-CoV-2 and related biomarkers [18]. Apart from this, nanotechnology can be used to develop new antiviral drugs, promote codelivery of multiple drugs, enhance circulation time and achieve sustained release of drugs. Moreover, it can also be used for pulmonary targeting, which can reduce the side effects of drugs [33]. Further, as this disease is too infectious to be controlled by just the above strategies, it requires the development of a vaccine. Here, nanotechnology can be used as a delivery agent for mRNA and DNA vaccines as a means of protecting them from enzymatic degradation, thus overcoming the bottlenecks in their *in vivo* application [17]. The following sections of this review aim to describe the application of nano-sized materials for prevention, early diagnosis of infection and treatment modalities for people infected with COVID-19 (Figure 2). This review may help in bringing forth the advantages of nanotechnology to make full use of its potential in overcoming this pandemic situation in the near future.

### Nanotechnology for preventing the spread of COVID-19

As COVID-19 is a highly contagious disease and presently there is a lack of effective treatment and vaccination for the same, preventing the spread of infection is of the utmost importance. Efforts have been made to prevent the transmission of the virus through social distancing, use of masks and PPE and reinforcement of hygiene methods [30]. In this context, several companies are investing in nanotechnology-based products for the development of effective cleaning products and PPE. Research evidence suggests that silver nanoparticles have potent antimicrobial effects and are one of the most useful metal disinfectants against viruses, bacteria and other micro-organisms [30,34]. Silver has been used to control infections since ancient times. The reported mechanisms for its antimicrobial activity are: inhibiting cellular respiration and disrupting metabolic pathways, leading to enhanced production of reactive oxygen species; forming pores and punctures on a bacterial cell wall by interacting with peptidoglycan molecules; and disrupting microbial DNA, thereby inhibiting viral replication [35]. Silver nanoparticles are better antimicrobial agents than their macro counterparts due to the larger surface-to-volume ratio that results from their nano size, which increases the area of reactivity with microbes and enhances cellular uptake and infiltration into biological membranes. Further, the toxicity of silver nanoparticles is size- and shape-dependent. It has been found that the smaller the size, the higher is the toxicity due to higher reactivity and ion release in cells [36].

Working in this direction SHEPROS, a Malaysian company, has developed Nano Silver sanitizer containing a suspension of silver nanoparticles of size 25 nm that kills a broad spectrum of micro-organisms, including viruses, by adversely affecting cellular metabolism and inhibiting cell growth through suppression of the basal metabolism of the electron transport system. This product is available on the market and can be used as a sanitizer against SARS-CoV-2 [37,38]. Similarly, Weinnovate Biosolutions, a startup supported jointly by the Indian Department of Science and Technology and Department of Biotechnology, has developed a nonalcoholic aqueous-based colloidal silver solution which shows its antiviral effect by preventing the synthesis of viral negative-strand RNA and viral budding [39]. Other silver-based nanoformulations marketed as sanitizers and disinfectants are listed in Table 1. NanoTouch Materials, LLC, a USA-based company, has developed NanoSeptic^®^ Surface, which helps in disinfection of public touchpoints, such as door handles, elevator buttons and even the rear of phones, protecting them against SARS-CoV-2. This disinfectant is composed of mineral nanocrystals, which act as a catalyst in the presence of light to create a powerful oxidation reaction that oxidizes organic contaminants [40].

**Table 1.** Table showing nanoparticles used for prevention and detection of SARS-CoV-2 infection.

Name of product (company)	Type of nanoparticles	Function
**Disinfectants and sanitizers**		
Nano Silver sanitizer (SHEPROS)	Silver nanoparticle (suspension)	Hand sanitizer (kills 99% of germs and bacteria)
Silvo Clean Spray (Weinnovate Biosolutions)	Silver nanoparticle (colloidal solution)	Sanitizer and disinfectant
NanoSeptic (NanoTouch Materials, LLC)	Mineral nanocrystal (creates an oxidation reaction, continuously oxidizing organic contaminants)	Surface disinfectant
TeqAir 200 air ionizer (TEQOYA)	–	Air purifier
AAVI Leaf^®^ (AAVI Technologies Co.)	–	Air purifier
Mack Antonoff HVAC (Mack Antonoff HVAC)	–	Air purifier
Air Decontamination Units (Genano Ltd)	–	Air purifier
**PPE**		
Graphene mask (Flextrapower, Inc.)	Graphene nanomaterial	Virus protective respiratory mask
Guardian G-Volt (LIGC Applications Ltd)	Graphene nanomaterial	Virus protective respiratory mask
G + Fibrics (Directa Plus PLC)	Graphene nanomaterial	Antiviral fabric is used in the production of medical devices such as masks, gloves and gowns to ensure better prevention against the spread of virus
Antiviral fabrics (Promethean Particles Ltd)	Copper nanoparticle (with Promethean particles)	PPE
MVX Nano Mask™ (MVX Prime Ltd)	–	Self-sanitizing surgical mask proven to kill 99.9% of all viruses and bacteria that come into contact
ReSpimask^®^ VK (RESPILON)	Copper oxide nanoparticles	Face mask with a 99.9% filtration efficiency for viruses; also inactivates the virus
Nanofiber mask (YAMASHIN-FILTER CORP.)	Nanofibers made from synthetic polymers	Respiratory protective mask
NANOHACK (Copper 3D Antibacterial Innovations)	Copper oxide nanoparticles	Protective respiratory mask
**Detection and diagnosis**		
COVID-19 Rapid POC CE-IVD Test (NanoComposix)	Gold nanoparticles	Has sensitivity and reliability of visual detection so used in point-of-care tests (detection kit)
COVID-19 Rapid Test Cassette (SureScreen Diagnostics Ltd)	Gold nanoparticles	Detection kit
COVID-19 point-of-need diagnostic test (Mologic Ltd)	Gold nanoparticles	Detection kit
SAFER-sample Kit (Lucence Diagnostics Pte Ltd)	–	Sample collection kit which stabilizes viral RNA at room temperature for a week which facilitates more accessible testing of viral infections such as COVID-19
Lateral flow (Sona Nanotech, Inc.)	Gold nanorod	Detection kit

HVAC: Heating, ventilation and air conditioning; POC: Point of care; PPE: Personal protective equipment.

All the data mentioned in this table were obtained from the Nanotechnology Product Database https://statnano.com/.

Studies suggest that surface contamination plays a significant role in the transmission of viruses. Several nanomaterials (e.g., titanium dioxide, copper oxide and silver nanoparticles), when associated with polymers and textiles, can reduce the viability of viruses on surfaces, especially in conditions of light exposure [41]. Working in this direction a Chilean/USA-based company, Copper 3D, has developed a nanocomposite face mask named NanoHack in which 5% copper oxide nanoparticles are impregnated in three layers of nonwoven polypropylene filters, bestowing them with excellent antiviral activity against SARS-COV-2. This mask is popular throughout the globe [42]. Promethean Particles Ltd, a UK-based company, is developing copper nanoparticle-embedded polymer fibers in collaboration with leading research facilities and textile companies for use in PPE kits [43]. Further, the development of protective materials that can not only capture the viruses but also kill them would have a far-reaching effect in preventing the spread of COVID-19. For this, nanomaterials that have an inherent antiviral activity, such as silver nanoparticles, graphene oxide (GO), copper oxide nanoparticles, two-dimensional carbides and nitrides can be employed. It was found that coating these nanomaterials on masks and PPE enhances their ability to capture and inactivate viruses [18]. In this context, RESPILON Group, a Czech Republic-based company, has developed ReSpimask^®^ VK, which is available on the market and has 99.9 % filtration efficiency for viruses and bacteria. The filter of the mask is enriched with accelerated copper oxide nanoparticles, due to which it not only intercepts the viruses but also actively kills them [44]. Further, a reusable and recyclable mask can also be developed by depositing a few layers of graphene on a low-melting-temperature unwoven mask. The excellent hydrophobic and photothermal properties of graphene help to repel the incoming aqueous droplets and allow for sunlight sterilization, respectively [45]. This product is still under development and is not yet available on the market. Apart from its usage in cleaning products and PPE, nanotechnology has also been employed in the development of air purifiers to prevent airborne transmission of the SARS-CoV-2 virus. In this context, the TeqAir 200 air ionizer developed by TEQOYA, a France-based company, is already on the market. As the size of SARS-CoV-2 is close to the median of the particle sizes for which TEQOYA air purifiers are efficient, they would be expected to reduce the concentration of SARS-CoV-2 in the air [46]. Apart from those mentioned above, we have summarized other examples of nanotechnology-based products to prevent COVID-19 spread in Table 1.

Past global experiences of viral outbreaks suggest that immunizing individuals is the only prevention from the future influence of viral infections, hence biomedical intervention toward vaccination is the prime focus of research. Vaccination has served as the most effective public health program that can prevent or control the spread of contagious disease and it seems to be the only hope to combat COVID-19. The uncontrolled increase in the number of SARS-CoV-2 infected cases and the emergence of new strains of SARS-CoV-2 has emphasized the urgent global need for vaccine development. Vaccination is the process of immunization whereby the host immune system is activated to induce long-term immune memory, which protects against future infection by a pathogen. It prevents infectious diseases by inducing a controlled immune response against the pathogen by mimicking its natural interaction with the host immune system. Vaccines consist of two major components: an antigen, which targets the immune system to activate it, and an adjuvant, which is coadministered with vaccines to potentiate or modulate the immune system against the antigen [47].

Conventional vaccines include either live attenuated pathogens which have a risk of reversion to virulent strains, or inactivated pathogens which generally display weak immunogenicity. This has led to the development of next-generation subunit vaccines like RNA or DNA encoding viral antigens, which could overcome these limitations; however, they suffer from low immunogenicity [48]. Because all of these are proteins which are easily degraded in the body, successful vaccines are still difficult to achieve for various infectious diseases. Nanotechnology-based platforms have been used for specific delivery and sustainable release of antigens, adjuvants and immunoregulatory agents [49] as they can control improper immune stimulation, loss of bioactivity of immunoactive agents during circulation, and off-target side effects. Pharmaceutical companies are using nanoparticles for vaccine development and delivery (Table 2). Companies like BioNTech/Pfizer and Moderna have encapsulated their mRNA vaccines in lipid nanoparticles [50,51], whereas Oxford/AstraZeneca and CanSino [52] have incorporated the antigen-encoding sequence into the DNA of adenovirus [53]. On the other hand, Novavax, Inc., a nanotechnology-based company, has conjugated the S protein of SARS-CoV-2 onto the surface of nano-sized virus-like particles [54] for effective delivery of vaccines to the host body [55]. The next-generation vaccines like subunit vaccines rely on adjuvants that can enhance the vaccine’s potency in elevating the immune response against specific antigens. In this regard, the nano-scale adjuvant can be of great potential in encapsulating and presenting these antigens to the immune cells to improve the immunogenicity in groups that respond poorly to vaccines [56]. Clinically relevant vaccine adjuvants like aluminum-based nanoparticles have been studied for their dendritic cell (DC) cross-presentation efficiency and subsequent induction of cellular immunity. Aluminum adjuvants are known to promote strong default T helper 2 cell differentiation and antibody production through DCs but lack the ability to induce a T helper 1 cell immune response. This can be improved by the use of alum nanoparticles in combination with Toll-like receptor ligands to enhance the cross presentations by DCs [57]. Knudesen *et al.* compared and categorized different clinical-grade nanoparticle-based adjuvants like alum, MF59 (R), GLA-SE, IC31 (R) and CAF01 based on their immune profiles and protective efficacy to give insights for the rational development of next-generation vaccines for humans [58]. Recently, Novavax marked the entry of its coronavirus vaccine candidate NVX-CoV2373, which includes the company’s proprietary Matrix-M™ adjuvant, to clinical trials [59]. Thus, owing to the flexible nature of nanotechnology, nanoparticles can be engineered to strengthen immune stimulation with desired adjuvant activities.

**Table 2.** Clinical status of nanotechnology-based vaccines in COVID-19.

Name of vaccine	Type of nanoparticle	Type of vaccine	Function of nanoparticle in vaccine	Company	Phase	Ref.
mRNA-1273	Lipid nanoparticle (composed of proprietary ionizable lipid, SM-102 and three commercially available lipids: cholesterol, DSPC and PEG2000 DMG)	RNA vaccine	Acts as mRNA carrier for safe and efficient transport *in vivo*	ModernaTX, Inc.	Phase III	[65]
NVX-CoV2373	Virus-like nanoparticle (Virus-like nanoparticle vaccine with saponin-based Matrix-M™ adjuvant)	Protein subunit vaccine	Thermostable, has a higher binding affinity toward the human ACE2 receptor and neutralizes virus infection	Novavax	Phase I/II	[66,67]
BNT162	Lipid nanoparticle (contains an ionizable cationic lipid/phosphatidylcholine/cholesterol/PEG-lipid [50:10:38.5:1.5 mol/mol])	mRNA vaccine	Acts as mRNA carrier for safe and efficient transport *in vivo*	BioNTech/Fosun Pharma/Pfizer	Phase I/II/III	[68–70]
ARCT-021	Lipid nanoparticle (contains ionizable lipid with a thioester to link the amine-bearing headgroup to lipid tails via two additional ester groups. Two possible ionizable lipids in this family are Lipid 10a or Lipid 2,2 4C CH3)	RNA vaccine	Acts as mRNA carrier for safe and efficient transport *in vivo*	Arcturus Therapeutics Ltd	Phase I/II	[71,72]
ChulaCov19	Novel lipid nanoparticles (Genevant LNP, likely cationic lipid CL_1_)	RNA vaccine	Acts as mRNA carrier for safe and efficient transport *in vivo*	Chulalongkorn University	Phase I	[72–74]
CVnCoV	Lipid nanoparticles (Acuitas LNP, possibly using the ionizable lipid ALC-0315)	mRNA vaccine	Acts as mRNA carrier for safe and efficient transport *in vivo*	CureVac AG	Phase III	[72,75,76]

DMG: Dimyristoyl glycerol; DSPC: 1,2-distearoyl-sn-glycero-3-phosphocholine; PEG: Polyethylene glycol.

All the data mentioned in this table were obtained from https://clinicaltrials.gov.

### Nanotechnology in the detection of SARS-CoV-2

COVID-19 patients exhibit a wide range of clinical symptoms that are similar to those of influenza and other respiratory diseases, thus accurate detection of disease is essential to initiate proper treatment and prevent the spread of infection [60]. Nucleic acid-based testing was initially the primary detection tool for SARS-CoV-2, whereby nasopharyngeal or oropharyngeal swabs are used for detecting the presence of virus using RT-PCR. However, this technique is time consuming and labor intensive and requires expensive instruments. As COVID-19 cases are continuously increasing, with almost 130 million people already affected across the globe by end of March 2021, the current situation demands the development of detection techniques that are rapid, cost-effective and easy to handle. Thus research should be centered on developing rapid, sensitive and accurate nucleic acid or protein-based tests and point-of-care testing (POCT) [61]. In this context, nanotechnology can greatly aid in enhancing the sensitivity of already available detection techniques like RT-PCR and immunofluorescence assays by virtue of the nanoparticles’ high surface-to-volume ratio, high adsorption, quantum size effects and high reactivity, which allows for efficient interaction with sample analytes. In addition, nanoparticles offer ease of surface functionalization, meaning that numerous ligands can be attached via covalent or noncovalent bonding, which further enhances selectivity and specificity and reduces the time of detection. Moreover, nanomaterials can also be employed as labels for enhancing the signals, which helps in achieving the detection of very low-magnitude signals. This nanolabeling can be done by attaching metal nanoparticles, such as silver or gold nanoparticles (Au-NPs) or quantum dots on targeted biorecognizing probes, which results in significant enhancement of signals [62–64]. Au-NPs have been incorporated in designing a wide range of virus detection tools due to their unique photonic, catalytic and electric properties, coupled with the molecular interaction specificity of various biomolecules such as antibodies, RNA aptamers and single-stranded DNA. In addition to this, they have excellent multiplexing capabilities, which further render them suitable for incorporation into state-of-the-art technologies for virus detection [77]. We have summarized nanotechnology-based diagnostic products available on the market in Table 1.

#### Nanotechnology-based nucleic acid tests

Loop-mediated isothermal amplification is a process similar to RT-PCR, but it requires a simple heat block rather than a complex machine, which reduces the cost significantly [78]. This technique, coupled with Au-NPs, has been used for the development of colorimetric assays for rapid detection of hepatitis E virus, offering high sensitivity and cost–effectiveness compared with RT-PCR detection [79]. A similar detection method could be developed for SARS-CoV-2 by designing specific primers for the virus. Silica-coated magnetic nanoparticles (Fe_3_O_4_/SiO_2_) have been used for detection of Hepatitis B (HBV) and Epstein–Barr viruses, where they were found to be more rapid and sensitive than commercialized kits like Dynabeads. This is probably because of the much larger surface area of Fe_3_O_4_/SiO_2_ nanoparticles as compared with Dynabeads, due to which it requires 15–20 s to attract these nanoparticles as compared with 2–3 min for Dynabeads using magnets. Further, as the surface area for these nanoparticles is higher, they can isolate DNA in samples with low virus concentrations [80]. Given that using these types of nanoparticles can possibly reduce the time required as well as increase the sensitivity, similar nanoparticles could be developed for isolation of RNA and detection of SARS-CoV-2. Two-dimensional gold nanoisland-based dual-functional plasmonic biosensors that integrate the plasmonic photothermal effect and the localized surface plasmon resonance sensing transduction technique have been developed for detection of SARS-CoV-2 [81]. Here, gold nanoislands functionalized with complementary DNA receptors are used to detect hybridized cDNAs of SARS-CoV-2. Because this technique uses two different angles of incidence, the plasmonic resonances for the plasmonic photothermal effect and localized surface plasmon resonance can be excited at two different wavelengths, which significantly enhances the sensitivity, stability and reliability compared with RT-PCR. Further, it can accurately discriminate similar sequences such as RNA-dependent RNA polymerase genes from SARS-CoV and SARS-CoV-2. However, the technique has yet not reached the market and is still in a developmental stage.

#### Nanotechnology-based protein tests

Antigens and antibodies generated in response to SARS-CoV-2 infection can be useful tools for diagnosis and surveillance of COVID-19 because they can be used to detect infected patients as well as recovered patients. In this context, UK-based SureScreen Diagnostics Ltd has developed its COVID-19 Rapid Test Cassette, which is available on the market for detection of SARS-CoV-2 infection. This assay integrates Au-NPs with lateral flow devices: Au-NPs embedded in the nitrocellulose test strip detect COVID-19 biomarkers (IgG and IgM), which are released on interaction with antibodies embedded in the strip, eliciting a color change [82]. Another nanotechnology-based approach for detection of COVID-19 involves the development of graphene-based field-effect transistor biosensing devices. These sensors are coated with specific antibodies against SARS-CoV-2 spike protein and can detect the spike protein at concentrations of 1 fg/ml in phosphate-buffered saline and 100 fg/ml in clinical transport medium, thus providing a highly sensitive, rapid and on-site detection method [83]. This technique is still in developmental stages.

#### Nanotechnology-based POCT

POCT can be utilized to diagnose infections in remote areas and provide instant care, which could assist in preventing the spread of infection. Further, data recorded can be transferred easily to central repositories, thus reducing the burden on central facilities. NanoComposix, a USA-based company, has developed a COVID-19 Rapid POC CE-IVD test, which integrates Au-NPs with lateral flow devices, as discussed above. This kit is available on the market for *in vitro* diagnosis [84]. Furthermore, systems that enable battery-operated excitation and capturing of emission signals via a smartphone camera can also be used for creating an effective and sensitive POC test [85]. Barcodes are designed to label different biomarkers to detect multiple analytes in one reaction tube using a sample from a single patient. Kim *et al.* have developed a quantum dot-based barcode assay for HBV infection demonstrating 91 and 95% clinical specificity and sensitivity, respectively, in diagnosing HBV after isothermal reverse polymerase amplification [85]. These could be tailored to develop a diagnostic method for the detection of SARS-CoV-2.

### Nanotechnology in the treatment of COVID-19

Presently, there is no specific drug recommended to prevent or treat COVID-19. However, specific treatments are under investigation, like remdesivir, which could be administered to patients diagnosed with severe disease [86–88]. This drug has been authorized by the US FDA for emergency use for the treatment of suspected or laboratory-confirmed COVID-19 in adults and children hospitalized with severe disease [89]. COVID-19 adversely affects the respiratory system, so it would be beneficial to conduct research in the field of cytokines that protect the respiratory system and promote lung homeostasis during viral infections. One such important cytokine is leukemia inhibitory factor, which modulates severe adverse events during acute respiratory distress syndrome. Though they have not been studied in COVID-19, leukemia inhibitory factor nanoparticles have shown clinical importance in animal models of autoimmune encephalomyelitis [90]. These inhalable nanoparticles exert immunomodulatory effects and increase tolerance in acute respiratory distress syndrome. These results suggest that they could also play a protective role in SARS-CoV-2 infection.

Blocking entry of the virus into the host is a successful strategy in several viral infections. As previously described, the virus enters into the host via interaction of the receptor-binding domain of its spike protein with ACE2; thus any drug which could disrupt the binding of SARS-CoV-2-RBD to ACE2 has the potential to inhibit the virus. Working in this direction, Zhang *et al.* have chemically synthesized SBP1, a 23-mer peptide fragment of the ACE2 peptidase domain α1 helix composed entirely of proteinogenic amino acids, which specifically and strongly binds to the SARS-CoV-2 spike protein, thus inhibiting the virus [91]. This novel peptide-based drug appears potent, but the delivery of such drugs is very challenging because enzymes in the body rapidly degrade them, reducing their efficacy. For this reason, the authors collaborated with researchers from Northwestern University’s Simpson Querrey Institute for delivery of the peptide drug in nanostructures having water-filled channels that are prepared by ‘gluing’ millions of peptides. The similarity in the chemical composition of the drug and the carrier allows for the development of nanostructures that could protect the peptide drug from enzymatic degradation while it circulates in the body. These peptide nanostructures against the SARS-CoV-2 spike protein are in the preclinical trial stage [92]. Apart from this, some studies report that nanomaterials can bind to viral particles and prevent their interaction with the host cell. For example, carbon quantum dots prevent the entry of another human coronavirus (HCoV-229E strain) into the host cells by interacting with the S protein of the virus, which is then not available to bind with the host cells. These nanomaterials may also prove beneficial in inhibiting the entry of SARS-CoV-2 in the host [93]. Once the virus has entered the host, it is essential to contain the virus; thus inhibiting viral replication is also an important antiviral strategy. Several nanomaterials exhibit an intrinsic ability to inhibit viral replication, such as Ag_2_S nanoclusters and ZnO nanoparticles. These are reported to modulate the host immune response to enhance the secretion of antiviral cytokines and suppress inflammation. Exploring these types of nanomaterials in COVID-19 may result in an effective therapeutic response [93].

Because the respiratory tract is the main target for SARS-CoV-2 infection, airborne nanoparticles can be used for direct pulmonary delivery, which offers the benefits of rapid absorption due to high vascularization and circumvention of the first-pass metabolism effect [18]. Drug delivery of nanoparticles to the lungs depends primarily on particle size, surface area, electrical charge and surface morphology of the nanoparticles; given that all these properties can be tailored by using nanoparticles, they can be used to cross mucosal membranes through the transmucosal route using endocytosis, carrier-mediated or receptor-mediated transport processes. Several nanoformulations, such as solid lipid nanoparticles, polymeric nanoparticles and liposomes, have been evaluated for various pulmonary diseases. These are coupled with devices such as nebulizers, pressurized metered dose inhalers, dry powder inhalers and soft mist inhalers for their delivery [94]. Working in this direction, Bioavanta LLC/Bosti Trading Ltd has developed Novochizol, an aerosol based on chitosan nanoparticles. This formulation strongly adheres to lung epithelial tissues and ensures sustained release without systemic distribution, making it an ideal intrapulmonary delivery system. Due to the above properties, the company is in the process of developing drug-loaded Novochizol for the treatment of COVID-19, and this is in the preclinical evaluation stage [92].

### Nanotechnology in immunomodulation

**Figure 3.**
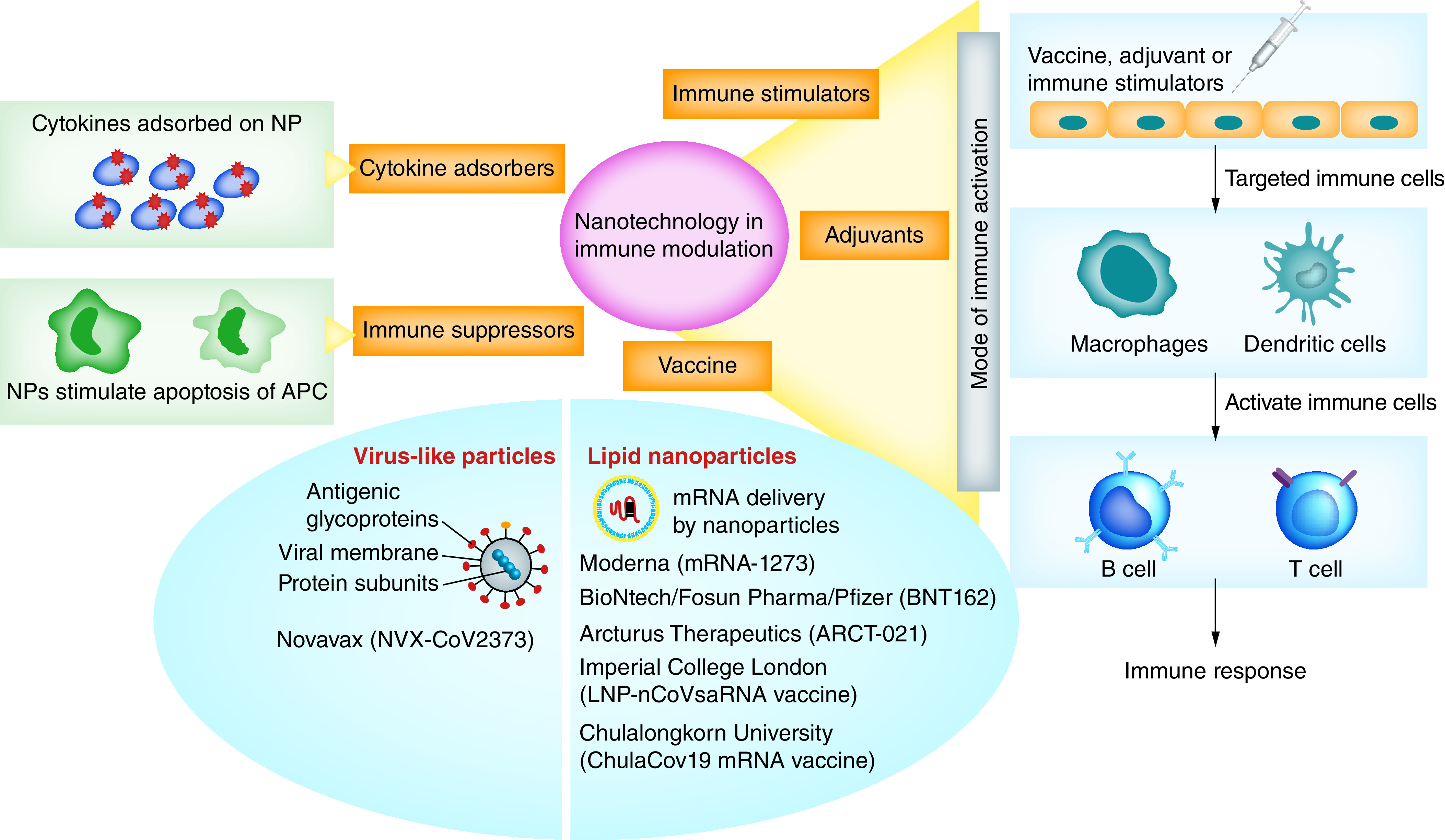
Role of nanoparticles in immune modulation. Nanoparticles can act as immune stimulators, adjuvants or vaccines that activate the host immune system. When these NPs are administered in the host body, they activate macrophages and dendritic cells and further activate B cells and T cells to generate both humoral and cell-mediated immune responses against the pathogen. Research is focused on either virus-like NPs or lipid NPs for the delivery of vaccines. NPs can also act as immune suppressors by stimulating apoptosis of APCs, such as macrophages and dendritic cells. NPs are also known for their capacity to adsorb cytokines on their surface to control cytokine storm in SARS-CoV-2 infected patients. APC: Antigen-presenting cell; NP: Nanoparticle.

Recent advances in designing nanomaterials that can directly modulate the immune system through their physical and chemical properties or deliver vaccines are novel approaches toward promoting immune responses against SARS-CoV-2 infection. Nanotechnology platforms have emerged as promising tools for the prevention and treatment of various infectious diseases by modulating the immune system, either by immunostimulation or immunosuppression [18]. Nanoimmunity-by-design possibly combines the safe-by-design concept, which is based on implementing the knowledge of physicochemical properties of nanomaterials in a structured way to develop safe nano-enabled products, and an immunity-by-design concept that functionalizes nanomaterials to fine-tune their physicochemical properties so that it can potentially modulate the immune system [95–97]. In the context of SARS-CoV-2, the application of these immunomodulatory nanosystems may be feasible to offer the development of novel antiviral therapeutics and vaccines (Figure 3) [49].

The understanding of immune responses toward nanomaterials is an important factor in designing biocompatible nanomedicines. As the immune system senses pathogens, it can also sense the nanomaterials by the chemical functional groups engineered on their surface. Immune sensing is also a crucial part of the development of vaccines [98]. Carbon and carbon-based nanomaterials such as graphene and nanodiamonds are sensed by immune cells and their interaction with immune cells elicits either an immune stimulating or suppressing response [96]. Orecchioni *et al.* studied the effects of GO and GO functionalized with amino groups (GONH_2_) on human immune cells. They observed that GONH_2_ polarized T cells and activated monocytes toward a T helper-1/M1-mediated immune response with low systemic toxicity [99]. Different surface functionalization of nanodiamonds showed pronounced regulation of immune-modulatory transcripts and enhanced the immunological compatibility [100]. Polypropylene sulfide vaccine nanoparticles have been engineered for complement activation and their functionalization is associated with antigen-specific immune responses that induced antibody production, T cell proliferation and IFN-γ cytokine production upon antigen restimulation [101]. Moreover, it has been reported that nanoparticles induce NLRP3-mediated inflammasome activation when internalized by antigen-presenting cells (APCs). The sequence of events that follows involves inflammasome complex formation with subsequent production of interleukins and activation of immune cells [102]. Another approach of stimulating immune cells like T cells involves the surface functionalization of nanoparticles with ligands that target specific APCs such as DCs, where the APC-targeting nanoparticles are capable of inducing both cellular and humoral responses. Uto *et al.* developed Ag-carrying biodegradable poly(γ-glutamic acid) (γ-PGA) nanoparticles that induced immune responses *in vivo*. The γ-PGA nanoparticles induced cytokine production, upregulated costimulatory molecules and enhanced T cell stimulatory capacity in DCs [103]. These nanoparticles could be explored to modulate the immune system of SARS-CoV-2-infected patients.

Studies have shown abnormal immune responses in SARS-CoV-2-infected patients with moderate and severe disease, whereby macrophages, neutrophils and inflammatory cytokines accumulate in the bronchoalveolar lavage fluid [104]. This massive cytokine storm reflects the uncontrolled deregulation of the host immune defense, which limits the understanding of immune signaling pathways associated with SARS-CoV-2 infection [28]. Nanomaterials can be exploited to control the cytokine storm in COVID-19 patients. The biomimetic macrophage-like nanoparticles that possess an antigenic exterior identical to that of macrophages have been used for cytokine sequestration because of their capability to bind to proinflammatory cytokines. This strategy of detoxification may provide a treatment option for suppressing cytokines and improving the clinical outcome for COVID-19 patients [105]. Zheng *et al.* demonstrated that graphene nanoplatelets rapidly and significantly removed proinflammatory cytokine markers from human plasma. The material was less cytotoxic and showed faster adsorption as compared with other carbon-based materials. They also developed a flexible freestanding graphene nanoplatelet–poly(tetrafluoroethylene) composite material with a high accessible surface area for targeted adsorption of cytokines [106]. Hence the intrinsic properties of nanoparticles can be manipulated as an immunomodulatory component for suppressing the immune system and their modulation toward immune cells to evoke an immune response in SARS-CoV-2 infection.

## Rethinking nanotechnology against COVID-19

During this state of global emergency created by the COVID-19 pandemic, humans are facing unprecedented challenges. Developments in nanomaterials are promising huge possibilities in healthcare, but the potential risks of nanoparticles for both public health and the environment cannot be ignored. Despite having many advantages, nanotechnology suffers from certain pitfalls that may have mild to adverse consequences in clinics for COVID-19 patients, which have been inadequately reviewed. For COVID-19 patients, inhalation of lipid-based nanomedicines could be beneficial as this method can deliver drugs directly into the lungs, bypassing first-pass metabolism. However, it can encounter obstacles like variation in drug absorption due to changes in nasal physiology, difficulty in crossing the nasal epithelium and, most importantly, lack of knowledge of nasal drug absorption [107,108]. Studies have also demonstrated a high pulmonary inflammatory response in the case of some nanoparticles. Moreover, due to the increased surface area to size ratio, many nanoparticles can interact with cellular macromolecules and lead to oxidative stress, which can have severe health impacts [109,110]. Besides absorption, digestion and systemic circulation, renal excretion of nanomedicines is an expected phenomenon in which the kidney plays a major role; hence nephrotoxicity of nanoparticles cannot be overlooked. Several studies on animal models have demonstrated that nanoparticles exhibit severe nephrotoxicity at both the tubular and the glomerular level, major signs of which are degeneration of tubular epithelial cells, renal interstitial fibrosis, swollen glomeruli, change in Bowman’s space and proliferation of mesangial cells [111].

To address such complex challenges upon application of nanotechnology for COVID-19, co-operation among a diverse range of researchers, doctors, pharmaceutical companies and regulatory boards is required. The present concerns of nanotechnology in this pandemic should be taken as an opportunity to reform nanoparticles to increase the safety to risk ratio. This can be achieved by proposing strategies to evaluate nanotoxicology profiles through standardized assay protocols at the early stages of clinical development and to study their potential risks in patients. Designing smart nanoparticles, like the formulation of stimuli-sensitive nanoparticles for controlled drug release, can also be beneficial to achieve site-specific drug targeting with reduced systemic toxicity [112]. Coating nanoparticles with polyethylene glycol is known to enhance their drug delivery capacity to the target cells, as PEGylation of nanoparticles can protect them from aggregation, opsonization and phagocytosis and prolong their systemic circulation [113]. Besides this, encapsulating cargo-loaded nanoparticles into bacteria, which are internalized by the mammalian cell and deliver the cargo specifically into the cell, can be an amazing strategy to deliver therapeutics or vaccines into COVID-19 patients [114]. Moreover, in-depth study and exchange of knowledge among different countries are essential to achieve a scientific solution for the fight against the coronavirus [115,116].

## Conclusion

In this review we have emphasized advances of nanotechnology and its clinical translation to counteract challenges related to the prevention, diagnosis, therapeutic delivery, vaccination, immunity generation and overall disease management of COVID-19. By now a lot is known about the life cycle, pathogenicity and the associated immune response of SARS-CoV-2, yet we lack strategies to prevent and treat the infection. Protection and diagnosis are important for preventing the spread of infection. Currently, drug repurposing is in high demand for therapy against SARS-CoV-2 infection, but is not effective in all cases. The only hope to control the pandemic situation is the development of an effective vaccine that can prevent the infection. In this regard, nanotechnology seems to be highly relevant due to the physicochemical properties of nanoparticles that provide an opportunity to fine-tune them against the SARS-CoV-2 infection. In this milieu, nanotechnology can play a big role in developing PPE and sanitizers which can help to prevent the transmission of infection. As the symptoms of COVID-19 are very similar to those of other respiratory diseases, it is important to have accurate, sensitive and rapid diagnostic tools to detect the infection at an early stage. In this context, it is reasonable to believe that due to their smaller size and larger surface area, nanotechnology products can detect the disease with high accuracy. With the growing demand for drug repurposing to find therapeutics against SARS-CoV-2 infection, it is equally important to develop nanoplatforms for effective delivery of the same to the target site. In addition to this, strategies are proposed whereby nanoparticles can themselves be used as therapeutics or immune-modulating agents upon SARS-CoV-2 infection. Given that vaccination gives high hope to combat COVID-19, the role of nanotechnology in the development and delivery of suitable vaccines and adjuvants is emphasized.

## Future perspective

Despite the advances achieved in understanding the molecular mechanisms of COVID-19, a thorough knowledge of viral transmission and behavior is still required to completely contain the infection. The outbreak of COVID-19 has forced the world to depend upon the science and research community to find viable solutions to this problem by advancing research toward basic sciences and translational studies. Advances in the design and fabrication of nanotechnology have made research more flexible and creative toward dealing with present challenges. Many nanoformulations have already been repurposed against the novel SARS-CoV-2 infection, signifying the potential of nanotechnology. Presently, many nanoformulations are on the market for disease prevention (hand sanitizers, disinfectants, masks, etc.), and we anticipate that broad-spectrum nanoformulations will be developed for vaccination and therapeutics that can be easily modified according to the need of the hour. As nanomedicine has shown promising results in infectious and noninfectious disease management, we believe that nanomedicines will also be able to withstand future pandemic situations arising from microbial infections. Thus there must be a structured clinical investigation and research empowerment in the field of nanomedicines with regard to infectious disease biology. Additionally, studies should be focused on the development of relevant models for evaluating the toxicity profile of these nanomedicines and their large-scale production for commercial use.

**Executive summary**

**Nanotechnology for preventing the spread of COVID-19**

- Silver nanoparticles have been used in successfully commercialized products as a disinfectant or sanitizer against SARS-CoV-2 due to their antimicrobial properties.
- Nanoparticles, such as titanium dioxide, copper oxide and silver nanoparticles can be blended in either polymers or textiles on the surface of personal protective equipment for protection against the virus.
- Lipid nanoparticles, adenovirus nanoparticles and virus-like particle nanoparticles are under clinical investigation for vaccine delivery to the host as they are capable of specific delivery and sustainable release of antigens, adjuvants and immunoregulatory agents.
- Alum, MF59 (R), GLA-SE, IC31 (R) and CAF01 are nanoparticle-based adjuvants which can enhance the efficiency of next-generation vaccines.

**Nanotechnology in the detection of SARS-CoV-2**

- Gold nanoparticles can be used for designing biosensors that can provide rapid and sensitive SARS-CoV-2 diagnosis tools.

**Nanotechnology in the treatment of COVID-19**

- Solid lipid nanoparticles, polymeric nanoparticles and liposomes can be coupled with nebulizers and inhalers for intrapulmonary delivery of therapeutics through the nasal route.

**Nanotechnology in immunomodulation**

- The immune system can sense nanoparticles through the functional groups engineered on their surface, which can be useful for the development of vaccines and therapeutics.
- Carbon-based nanomaterials such as graphene and nanodiamonds are sensed by immune cells, which elicits or suppresses the immune response, modulating the host immune system.

**Rethinking nanotechnology against COVID-19**

- For implementing nanotechnology in clinical practices in this current pandemic situation, strong collaboration among researchers, doctors, pharmaceutical companies and regulatory boards is mandatory.
